# Dorsal radial artery catheterization for invasive blood pressure monitoring

**DOI:** 10.5935/0103-507X.20200022

**Published:** 2020

**Authors:** Roosevelt Santos Nunes, Camila Mussolin Tamaki, Heloisa Helena Robles Penha, Julia Carvalho Mafra Terra, Geraldo Luiz de Figueiredo, Gil Cezar Alkmin Teixeira

**Affiliations:** 1 Hospital Unimed de Ribeirão Preto - Ribeirão Preto (SP), Brazil.

## To the Editor

Arterial catheterization is a common procedure in intensive care units, with the primary purposes of continuous blood pressure monitoring and frequent blood sample collection. Obtaining an arterial line is also critical for the use of minimally invasive hemodynamic monitoring.

The radial artery is often the first choice for the placement of an arterial catheter, mainly because of its superficial location. The conventional method for locating the radial artery is to palpate the pulse, taking into account the anatomical reference points. However, the position of the artery varies in 30% of patients.^(1)^ In addition, some factors make palpation of the artery difficult or even impossible: severe hypotension, morbid obesity, arterial scarring, edema, and atherosclerosis.^(2)^ The use of ultrasound in arterial catheterization facilitates locating the artery and catheter cannulation, which increases the success rate in the first puncture attempt, reduces the time for cannulation success, and, consequently, decreases the occurrence of complications.^(3,4)^

A high incidence of compromised blood flow has been reported, as detected by ultrasound after placement of the catheter in the radial artery,^(5)^ indicating that this procedure is subject to ischemic complications. Although the Allen test is a proven method for assessing collateral circulation, some studies have shown that it is not adequate for predicting complications in the placement of a radial arterial line.^(6,7)^ Evaluation of radial artery diameter by ultrasound to determine adequate catheter dimensions can reduce the risk of arterial occlusion related to radial cannulation. Another measure that can be adopted to minimize the risk of ischemic complications is distal puncture of the radial artery through the anatomical snuffbox.

The anatomical snuffbox is a triangular cavity in the dorsal region of the hand delimited medially by the tendon of the extensor pollicis longus and laterally by the extensor pollicis brevis and the abductor pollicis longus, with a floor formed by the scaphoid and trapezium bones. The digital arteries that supply the fingers arise from the superficial palmar arch. The palmar branch of the radial artery, which contributes to the superficial palmar arch, arises from the radial artery on the palmar side of the wrist before entering the anatomical snuffbox. The advantage of dorsal radial artery catheterization is that cannulation can be performed more distally - beyond the origin of the palmar branch - than when using the conventional approach, and it reduces the risk of digital ischemia.^(8)^ Another benefit is patient comfort, especially in those with orthopedic problems, which prevents supination of the arm - a necessary position in the conventional technique.^(9)^

According to this distal puncture technique, the hand is positioned so that the anatomical snuffbox is facing upwards. The artery is typically palpated at the intersection of the thumb with the first finger over the bone structures of the snuffbox. Once a pulse is detected, access is obtained by following the same recommendations of antisepsis and asepsis of conventional insertion. The radial artery is smaller in the dorsal region and may be less adequate for some patients.^(9)^ Ultrasound is a fundamental aid to assess the adequacy of vascular size and location.

As an example of the application of this technique, we report the case of a cannulation of the right dorsal radial artery in a patient who had digital ischemia in the contralateral hand related to the arterial line. The patient was a 73-year-old man with systemic arterial hypertension and prostate neoplasia who had been transferred from another hospital after prolonged hospitalization due to pneumococcal meningitis. The patient was tracheostomized at hospital admission and was dependent on mechanical ventilation and hemodialysis. Cultures were collected, and invasive devices were replaced. On the second day of hospitalization, the patient was diagnosed with septic shock. In two blood culture samples, there was growth of *Klebsiella pneumoniae* sensitive only to amikacin. Due to new hemodynamic instability, an arterial catheter was placed for invasive blood pressure monitoring.

**Figure 1:**
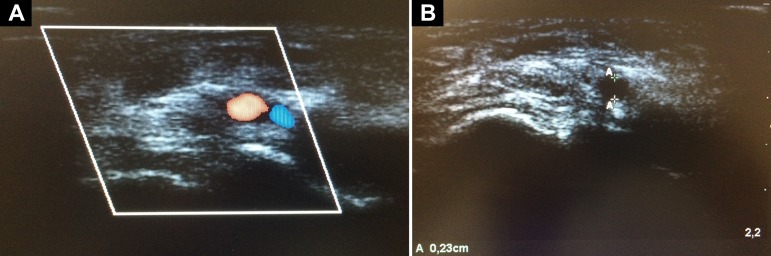
Ultrasonographic evaluation of the radial artery. (A) Cross-sectional view of the radial artery with color Doppler. (B) Measurement of the diameter of the dorsal radial artery.

**Figure 2:**
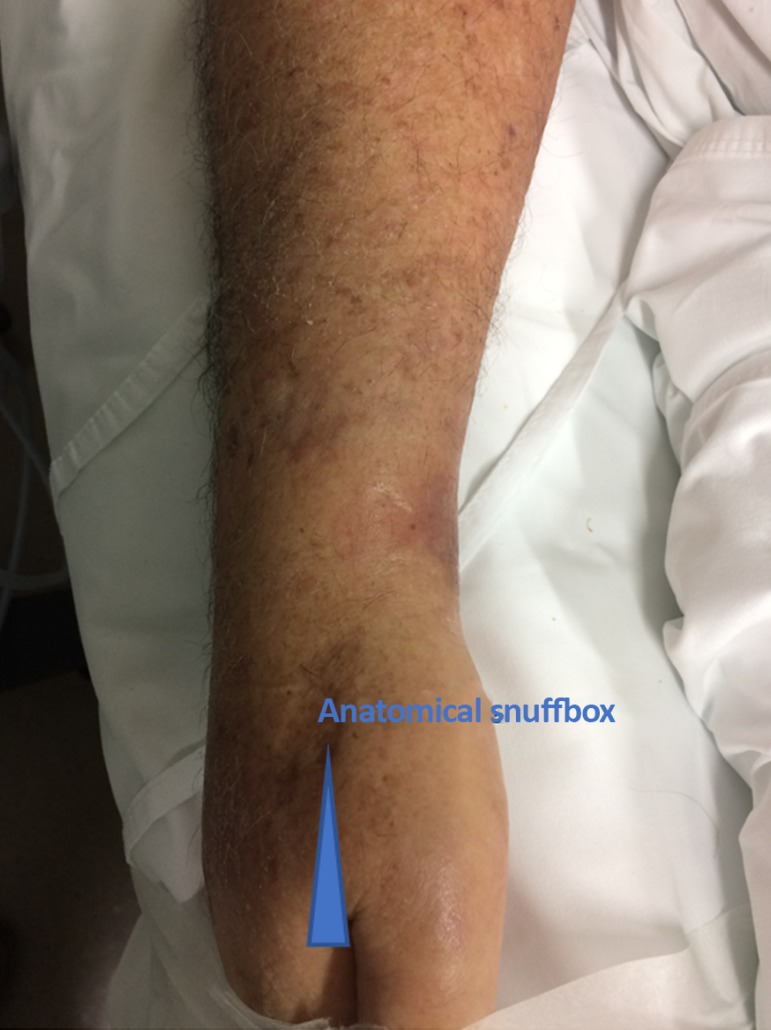
The patient’s arm is positioned so that the anatomical snuffbox is facing upwards.

**Figure 3:**
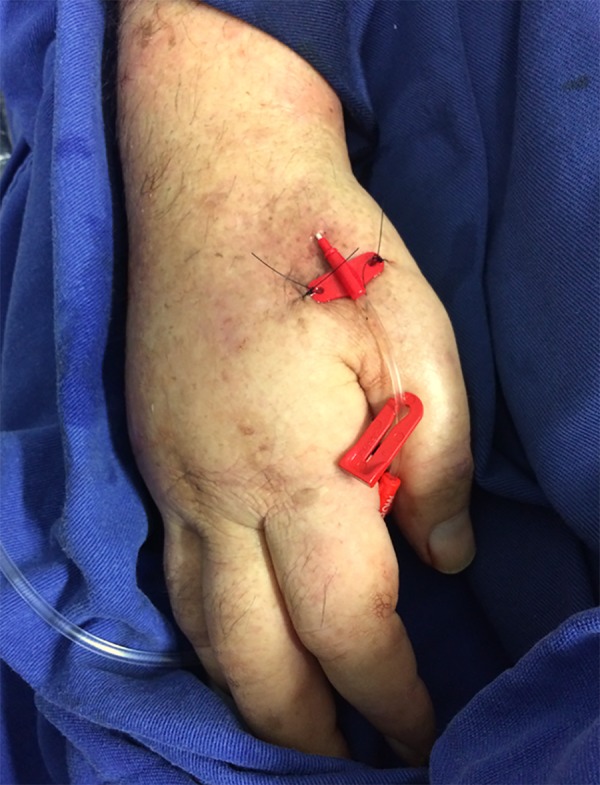
20-G arterial catheter inserted into the right radial artery by distal puncture (image obtained at the end of the procedure).

The patient was evaluated for the presence of pulse for puncture, and ultrasound examination was performed to assess patency and to measure the vessel diameter (Figure 1). A portable Sonosite M-Turbo ultrasound device (SonoSite Inc, Bothell, WA) equipped with a 13-6MHz linear transducer (HFL38x) was used. In the bed and with the headrest elevated to 30º, the patient had the right arm positioned in a way such that the anatomical snuffbox was facing upwards (Figure 2). Following infiltration of 3mL of lidocaine without vasoconstrictor into the radial fossa, the radial artery was punctured at an angle of 30°, and the catheter was gently implanted using the Seldinger technique (Figure 3). For this purpose, a 20-G x 8cm Seldinger arterial catheter (Arrow International Inc, Cleveland, OH) was used. The puncture was guided by ultrasound using the dynamic technique with a single-operator, and success was obtained on the first attempt. The catheter was fixed, and dressing was performed. The estimated time of the procedure was 15 minutes. The catheter remained for 8 days, without the occurrence of digital ischemia or any other complications directly related to arterial catheterization and was removed after septic shock resolved.

Radial dorsal access is an easy and safe intervention. In the intensive care setting, in which an arterial line remains for several days, this distal puncture technique may be a promising measure to minimize the risk of ischemic complications associated with the procedure. This technique has been widely used for coronary angiography, but thus far, there have been no reports in the literature on critically ill patients.
